# The anteroposterior axis of the tibia is adjusted to approximately a right angle to the anterior pelvic plane in the standing position in patients with hip dysplasia similar to normal subjects: a cross-sectional study

**DOI:** 10.1186/s13018-018-0816-z

**Published:** 2018-05-02

**Authors:** Norio Imai, Dai Miyasaka, Hayato Suzuki, Kazuki Tsuchiya, Tomoyuki Ito, Izumi Minato, Naoto Endo

**Affiliations:** 1 Division of Comprehensive Geriatrics in Community, Niigata University Graduate School of Medical and Dental Sciences, Niigata, 9518510 Japan; 2 Department of Orthopedic Surgery, Niigata University Medical and Dental Hospital, Niigata, 9518510 Japan; 3 Division of Advanced Materials Science and Technology, Niigata University Graduate School of Science and Technology, Niigata, 9502181 Japan; 4 Department of Orthopedic Surgery, Saiseikai Niigata Daini Hospital, Niigata, 9501104 Japan; 5 Department of Orthopedic Surgery, Niigata Rinko Hospital, Niigata, 9508725 Japan

**Keywords:** Anteroposterior axis of tibia, Tibiofemoral rotation, Femoral neck anteversion, Clinical epicondylar axis, Anterior pelvic plane, Lower extremity alignment

## Abstract

**Background:**

We previously described that the anteroposterior (AP) axis of the tibia is approximately perpendicular to the transverse axis of the anterior pelvic plane (APP) in the standing position in healthy subjects. The purpose of this study was to investigate the rotational alignment between the APP and clinical epicondylar axis and the AP axis of the tibia relative to pelvic coordination in the standing position in normal subjects and in women with developmental dysplasia of the hip (DDH) to aid decision making for surgeons in the alignment of implants in total hip or knee arthroplasty.

**Methods:**

This study included 77 Japanese women. Twenty-nine in the DDH group underwent curved periacetabular osteotomy; 48 women without lumbago and knee pain were included in the normal group. Femoral neck anteversion (FNA), condylar twist angle, and knee rotation angle were measured in femoral coordination. The angle between the femoral neck axis and clinical epicondylar axis (CEA) was measured, the transverse axis of the APP was also measured, and the angle between the AP axis of the tibia and transverse axis of the APP was calculated.

**Results:**

There was a moderate negative correlation between FNA and CEA relative to the APP. This finding indicated a trend towards greater FNA leading to more internal rotation. Knee rotation angle (KRA) relative to the APP was 1.65° ± 5.58° in the normal group and − 2.65° ± 7.57° in the DDH group. This finding indicated that the tibia AP axis was approximately perpendicular to the APP in the standing position both in the normal and DDH groups.

**Conclusion:**

We found that the tibia AP axis was at approximately a right angle to the transverse axis of the APP in the standing position in both the normal and DDH groups, while the KRA was different in the normal and DDH groups. These findings may prove helpful for positional alignment investigations needed for implantation in total hip or knee arthroplasty and gait analysis.

## Background

Spatial and geometrical malalignment between the femur and tibia such as tibiofemoral abnormal rotation is considered to cause some pathologies in the lower extremities, such as osteoarthritis of the knee and patellofemoral disorder including patellar dislocation, among others [1–7]. Moreover, Watanabe et al. [8] indicated that when preoperative rotational mismatch persisted, rotational mismatch could still occur after total knee arthroplasty, even if the components were placed in the correct position relative to anatomical landmark. Therefore, it is important to evaluate tibiofemoral rotation correctly.

Formerly, the alignment of the lower extremity was commonly evaluated with two-dimensional (2D) plain X-ray [7, 9, 10]. However, measurements with this 2D method are affected by the position of the pelvis and lower extremities of the subjects [11], and it is considered to produce measurement error and lead to reduced accuracy and reproducibility. Furthermore, the 2D method cannot assess rotational alignment such as tibiofemoral rotation.

Several reports have described that the anteroposterior (AP) axis of the tibia, which is defined by a line passing through the middle of the posterior cruciate ligament and the medial border of the patellar tendon attachment (so-called Akagi’s line), is at almost a right angle to the clinical epicondylar axis (CEA) [8, 12, 13].

We previously found that the CEA and the transverse axis of the anterior pelvic plane (APP) are approximately parallel in the standing position in healthy subjects [14]. Moreover, we also described that the AP axis of the tibia is approximately perpendicular to the transverse axis of the APP in the standing position in healthy subjects [15]. It is generally known that the proximal femur is more anteverted in patients with developmental dysplasia of the hip (DDH) [16, 17]. Consequently, it is speculated that the femur is more internally rotated during standing and walking in individuals with DDH compared to that in normal persons [18]. However, to our knowledge, no report has described the rotational alignment between the anterior pelvic plane (APP) and anteroposterior (AP) axis of the tibia in patients with DDH.

The purpose of this study was to investigate the rotational alignment between the APP and CEA, and the AP axis of the tibia relative to pelvic coordination in the standing position in normal subjects and women with DDH to aid surgeons in decision making for the alignment of implants in total hip or knee arthroplasty, as well as treatment for patellar dislocation and positional alignment investigations such as gait analysis.

## Methods

### Subjects

For this study, 77 Japanese women were enrolled because patients with DDH are reported to have a 9:1 female dominance [19]. Twenty-nine patients (29 legs) with bilateral DDH (mean age, 35.8 ± 8.8 years) from our institution who had undergone curved periacetabular osteotomy [20] for the early treatment of osteoarthritis of the hip joint due to acetabular dysplasia and whose center-edge angle of the hip joint was < 25°, as evaluated in the anteroposterior view on a plain radiograph of the hip, were enrolled in the DDH group. Patients with DDH who had previously undergone hip surgery or those with Crowe stages 2–4 of subluxation or Tönnis grades 2 and 3 of arthritic change according to plain radiographs of the hip bilaterally were excluded from this study. We also included 48 women (48 legs; mean age of women, 54.0 ± 10.8 years) without lumbago and knee pain and without any abnormal findings of the knee and spine on radiographic examination recruited from the family of outpatients and medical staff in the normal group. Computed tomography scans from all participants were examined to reconstruct a 3D bone model. With regard to the DDH group, computed tomography scans were examined before their operation to plan for osteotomy. This study was performed with the approval of the institutional research board of Niigata University Medical and Dental Hospital and written informed consent was obtained from the participants of the normal group. With regard to DDH group, the need for informed consent was waived because of the cross-sectional nature of this study that did not provide an intervention.

Radiographic examinations, including biplanar computed radiography images, were performed in the standing position, where each subject adopted a relaxed position with their knees fully extended and the toes aligned to the shoulders. Computed tomography from the pelvis to proximal tibia was also performed in the supine position with the knees fully extended.

### Measurements

**Fig. 1:**
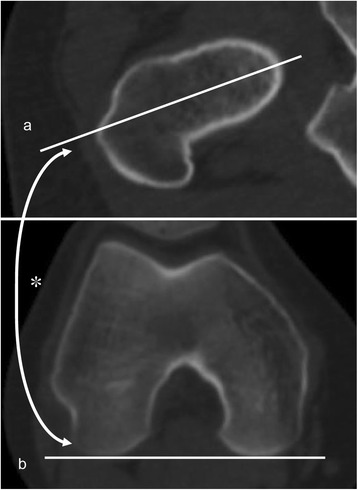
Measurement of the FNA. FNA (asterisk) is the angle between the femoral neck axis (a) and the PCA (b). FNA: femoral neck anteversion, PCA: positive external rotation

**Fig. 2:**
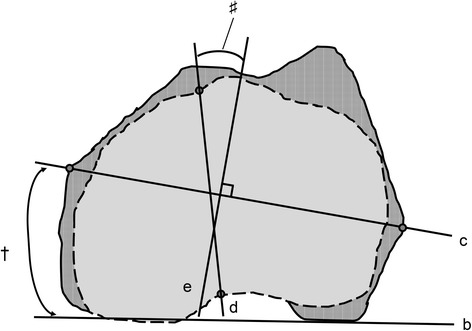
Measurement of the CTA and knee rotation angle. CTA (dagger) is the angle between the PCA (b) and the CEA (c). KRA (number sign) is the angle between the tibial AP axis (d) and the line perpendicular to the femoral CEA (e). The solid lines represent the contour of the projected femoral condyle onto the femoral horizontal plane. The dotted lines represent the contour of the projected tibial condyle onto the femoral horizontal plane. CEA: clinical epicondylar axis, CTA: condylar twist angle, KRA: knee rotation angle, PCA: positive external rotation

We used ZedHip® software (Lexi, Tokyo, Japan) to create three-dimensional (3D) digital bone models of the pelvis and femur and accurately reconstruct the spatial relationship between them [15, 21, 22]. We adjusted the 3D pelvis model to the APP [23], which contains both the anterior superior iliac spines and the pubic symphysis, which are the origin of this pelvis coordinate system. In the ZedHip® system, when the pelvis was adjusted to the APP, other bones such as the femur and tibia synchronously moved according to the pelvis position. With regard to the femoral coordinate system, the 3D model of the femur was positioned with the retrocondylar plane, which contains the bilateral posterior condyles and the most posterior point of the greater trochanter [24]. The femoral neck axis was defined as that in the method described by Sugano et al. [25] and measured in the plane just below the femoral head. Femoral neck anteversion (FNA) was measured as the angle between the femoral neck axis as above and the posterior condylar axis (PCA) (Fig. 1). Further, the CEA was defined as the line connecting the most prominent point of the medial epicondyle and the lateral epicondylar prominence. The condylar twist angle (CTA) was measured as the angle connecting the CEA and PCA (positive values indicate that the CEA is externally rotated relative to the PCA) (Fig. 2) [18]. The determination of the CEA and measurement of the CTA were also performed in the retrocondylar plane. The line through the midpoint of the lateral epicondylar prominence and the most prominent point of the medial epicondyle, and the line perpendicular to the CEA was defined as the femoral AP axis. With regard to the AP axis of the tibia, Akagi’s line [14] was selected. The knee rotation angle (KRA) was measured as the angle connecting the femoral AP axis and the AP axis of the tibia, projected onto the horizontal plane of the femoral coordinate system (Fig. 2). In the present study, negative values were defined as the internal rotation of the tibia relative to the femur and positive values as the external rotation.

**Fig. 3:**
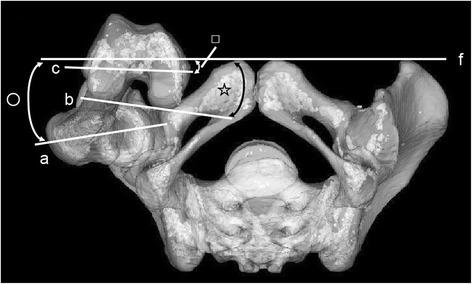
Measurement of the APP-FNA, APP-PCA, and APP-CEA. APP-FNA (white circle), APP-CEA (white square), and APP-PCA (white star) were defined as the angles connecting the FNA (a), PCA (b), and CEA (c), respectively, to the transverse axis APP (f). APP: anterior pelvic plane, CEA: clinical epicondylar axis, FNA: femoral neck anteversion, PCA: positive external rotation

FNA, CEA, and PCA relative to the APP were also measured in the standing position (APP-FNA, APP-CEA, and APP-PCA, respectively) using HipCAS® software (Lexi, Tokyo, Japan). The 3D digital bone models were projected onto the biplanar computed radiography images to match the contours of the 3D digital models with the computed radiography images for rotations and translations [15, 21, 22]. Kobayashi et al. [22] previously described the accuracy of HipCAS® in creating a 3D digital bone model that accurately reproduced the spatial relationship between the pelvis and the femur, and calculated the various alignment parameters within 1° and 1 mm of accuracy. Therefore, projection error and misalignment were estimated to be small in the current study. APP-FNA, APP-CEA, and APP-PCA were the angles that connected the FNA, CEA, and PCA projected onto the transverse plane of the pelvis and the APP was the line connecting both anterior superior iliac spines (Fig. 3).

**Fig. 4:**
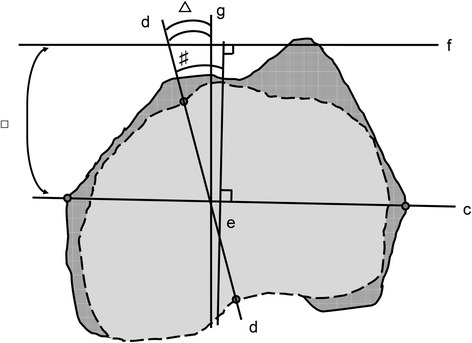
Calculation of the estimated AP axis of the tibia and the APP transverse axis. We calculated the estimated angle between the AP axis of the tibia (d) and the perpendicular line to the APP (g) from the KRA (number sign) and APP-CEA (white square) with the formula: (AP axis of the tibia relative to the APP transverse axis (white triangle)) = (APP-CEA) − (KRA). The solid lines represent the contour of the projected femoral condyle onto the femoral horizontal plane. The dotted lines represent the contour of the projected tibial condyle onto the femoral horizontal plane. AP: anteroposterior, APP: anterior pelvic plane, CEA: clinical epicondylar axis, KRA: knee rotation angle

Lastly, we calculated the estimated angle between the APP and AP axis of the tibia from the KRA in femoral coordination and APP-CEA with the formula: (AP axis of the tibia relative to the APP transverse axis) = (APP-CEA) − (KRA, the angle between the AP axis of the tibia and the line perpendicular to the CEA), as in our previous study [18] (Fig. 4).

### Statistical analysis

We used SPSS statistical software (SPSS version 24, Inc., Chicago, IL, USA) to analyze the data. Regarding the FNA, CTA, KRA, APP-FNA, and APP-CEA, we used Pearson coefficients to determine the correlation coefficients. To evaluate variation, we calculated the mean absolute difference (MAD), variability (standard deviation), and intraobserver reliabilities with intraclass correlation coefficients (ICCs) and a two-sided 95% confidence interval. We measured intraobserver reliability with two measurements by one observer at at least 1-week intervals. Moreover, we also compared the measurements to assess the interobserver reliability by a single measurement with two observers. A *p* value < 0.05 was considered statistically significant.

## Results

The details of the research subjects are demonstrated in Table 1. The FNA and KRA was significantly larger in the DDH group, while the CTA was almost the same (Table 2). There was a moderate positive correlation between the FNA and APP-FNA (regression equations: *y* = 0.32*x* + 5.57 in the normal group, *y* = 0.43*x* + 2.87 in the DDH group) and a negative correlation between the FNA and APP-CEA (regression equations: *y* = − 0.12*x* + 1.74 in the normal group, *y* = − 0.32*x* + 3.04 in the DDH group) (Table 3). This finding indicated a trend towards a greater FNA leading to more internal rotation. We also found a moderate positive correlation between the CTA and KRA in only the DDH group (regression equation: *y* = 1.51*x* − 9.57). This finding indicated a trend towards greater CTA leading to more external rotation of tibia relative to the femur in only the DDH group; there was no significant difference the between normal and DDH groups.

**Table 1:** Baseline characteristics of the participants

	Normal group (*n* = 48)	DDH group (*n* = 29)
Age (years)	54.0 ± 10.8***	35.8 ± 8.8***
Body height (cm)	153.6 ± 5.8	159.4 ± 6.8
Body weight (kg)	52.6 ± 7.6	55.5 ± 7.5
BMI (kg/m^2^)	22.3 ± 2.7	21.9 ± 2.9

Values are mean ± standard deviation

*BMI* body mass index

*p < 0.05, **p < 0.01, ***p < 0.001

**Table 2:** Measurement of anatomical and positional angles

	Normal group (*n* = 48)	DDH group (*n* = 29)
FNA (deg)	17.10 ± 9.16***	28.60 ± 12.69***
CTA (deg)	7.24 ± 1.89	7.37 ± 2.09
KRA (deg)	1.98 ± 6.86***	8.88 ± 7.02***
APP-FNA (deg)	10.73 ± 8.21*	16.71 ± 10.59*
APP-CEA (deg)	− 0.62 ± 4.24**	− 5.21 ± 8.55**
APP-tibia AP axis (deg)	1.65 ± 5.58*	− 2.65 ± 7.57*

Values are mean ± standard deviation

*AP* anteroposterior, *APP* anterior pelvic plane, *APP-CEA* clinical epicondylar axis relative to the APP, *APP-FNA* FNA relative to the APP, *CTA* condylar twist angle, *FNA* femoral neck anteversion, *KRA* knee rotation angle

*p < 0.05, **p < 0.01, ***p < 0.001

**Table 3:** Correlation coefficient between each parameter in the normal and DDH groups

	FNA (deg)	CTA (deg)	KRA (deg)	APP-FNA (deg)	APP-CEA (deg)	APP-tibia AP axis (deg)
FNA (deg)		0.085	0.130	0.421*	− 0.352*	− 0.009
		0.365	0.319	0.433*	− 0.451*	− 0.176
CTA (deg)			− 0.211	0.142	0.176	0.096
			0.505*	0.205	− 0.042	0.338
KRA (deg)				0.086	− 0.218	− 0.214
				0.107	− 0.181	0.266*
APP-FNA (deg)					− 0.396*	− 0.077
					0.410*	0.226
APP-CEA (deg)						0.048
						0.375*

Upper row: normal group; lower row: DDH group

*AP* anteroposterior, *APP* anterior pelvic plane, *APP-CEA* clinical epicondylar axis relative to APP, *APP-FNA* FNA relative to APP, *CTA* condylar twist angle, *FNA* femoral neck anteversion, *KRA* knee rotation angle

*p < 0.05

The KRA relative to the APP was 1.65° ± 5.58° in the normal group and − 2.65° ± 7.57° in the DDH group (Table 2). This finding indicated that the tibia AP axis was approximately perpendicular to the APP in the standing position.

Regarding validation, we obtained a high ICC for both intraobserver and interobserver reliability (Table 4).

**Table 4:** Intra- and interobserver reliabilities of each parameter

	Intraobserver reliability		Interobserver reliability	
	MAD ± SD	ICC	MAD ± SD	ICC
FNA (deg)	1.28 ± 1.45	0.908	1.55 ± 1.82	0.858
CTA (deg)	0.64 ± 0.48	0.936	0.79 ± 0.57	0.918
KRA(deg)	1.47 ± 1.72	0.861	1.87 ± 1.84	0.829
APP-FNA (deg)	1.57 ± 1.86	0.818	1.78 ± 1.93	0.806
APP-CEA (deg)	0.73 ± 0.52	0.937	0.86 ± 0.77	0.914
APP-tibia AP axis (deg)	1.24 ± 0.92	0.868	1.58 ± 1.34	0.857

*AP* anteroposterior, *APP* anterior pelvic plane, *APP-CEA* clinical epicondylar axis relative to the APP, *APP-FNA* FNA relative to the APP, *CTA* condylar twist angle, *FNA* femoral neck anteversion, *ICC* interclass correlation coefficient, *KRA* knee rotation angle, *MAD* mean absolute difference, *SD* standard deviation

## Discussion

In this current study, we evaluated the spatial relationship between the tibia AP axis and APP during standing in normal subjects and patients with DDH.

We found that the mean value of the KRA was 2.06° in the normal group. This value is similar to that obtained in previous reports for normal participants [14–16]. Conversely, in patients with DDH, the KRA was 8.88°, indicating that the tibia AP axis was not nearly at a right angle to the CEA; instead, it was externally rotated relative to it. Nevertheless, the KRA relative to the APP was almost perpendicular to the APP in the standing position in both groups (1.65° in the normal group and − 2.65° in the DDH group).

Parikh et al. previously described that the medial rotation of the femur attributed to increased femoral anteversion leads to increased compensatory lateral rotation of the tibia, resulting in abnormal patellofemoral loads, and increased compression of the lateral patella facet and tension of the medial patellofemoral ligament; these biomechanical alterations consequently caused a tendency for anterior knee pain and/or lateral subluxation of the patella [26]. In patients with DDH, the femur is generally more anteverted and tibia is externally rotated relative to the femur; therefore, anterior knee pain and/or lateral subluxation of the patella may be more likely to occur than in normal persons. Moreover, surgeons should be mindful of mistracking, subluxation, and/or dislocation of patella in patients with DDH who undergo total knee arthroplasty.

We previously described that the CEA, considered as the functional flexion-extension axis of the knee [27, 28], was approximately parallel to the transverse axis of the APP plane in the standing position in normal subjects [14]. Moreover, we also reported that the AP axis of tibia was at approximately a right angle to the transverse axis of the APP plane in the standing position in normal subjects [18]. In our current study, we found that the tibia AP axis was approximately perpendicular to the APP, while the KRA was significantly larger in the DDH group. These results in our studies may be important to integrate these two axes with regard to not only the anatomical reference, but also the kinesiology. They may also prove helpful to decide the alignment of implants in total hip or knee arthroplasty, treatment for patellar dislocation, and positional alignment investigation such as gait analysis. However, further examination is required with regard to knee motion in patients with DDH because rotation between the tibia and femur during flexion and extension are the same in normal persons and patients with DDH.

The current study has several limitations. First, only a few subjects and only middle age people were enrolled in the normal group. Therefore, we cannot perform a power analysis. Second, the KRA was examined in the supine position, while the APP-FNA and APP-CEA were examined in the standing position. However, according to several reports, the difference in KRA between the supine and standing positions appears negligible [15, 16]. Kozanek et al. stated that the KRA was approximately 3° at contralateral toe-off, and nearly 0° from ipsilateral heel-rise to contralateral heel-strike, respectively, during the stance phase of treadmill gait [29]. Third, there was a difference in the plane of the measurement; the KRA was examined in femoral coordination, while the APP-FNA and APP-CEA were examined in pelvis coordination. Chen et al. reported that the tibia was internally rotated approximately 3° relative to the femur when the knee was flexed from 0° to 8° [30]. We preliminarily measured the femur in the 5° flexion and 3° adduction relative to the APP in the standing position on average and our computer simulation found that, with the lower extremity in this position, the expected difference of the angle was not more than 0.5°. Therefore, we believe that the position of the lower extremity did not affect the results of this study. Fourth, we examined only women in this study, because patients with DDH are reported to have a 9:1 female dominance [19]. In our institution, patients with DDH who had undergone curved periacetabular osteotomy were less than 15 in number over 8 years. Similar examination is required in male subjects in the future.

## Conclusions

We found that the tibia AP axis was at approximately a right angle to the transverse axis of the APP in the standing position in both the normal and DDH groups, while the KRA was 8.88° in the DDH group. From our results, we believe that the femur is adjusted such that the anteroposterior axis of the tibia is approximately at a right angle to the anterior pelvic plane in the standing position not only in normal persons, but also in patients with hip dysplasia. These findings may prove helpful to decide the alignment of implants in total hip or knee arthroplasty, treatment for patellar dislocation, and positional alignment investigations such as gait analysis.

## Abbreviations

AP: Anteroposterior
APP: Anterior pelvic plane
CEA: Clinical epicondylar axis
CTA: Condylar twist angle
FNA: Femoral neck anteversion
ICC: Intraclass correlation coefficient
KRA: Knee rotation angle
MAD: Mean absolute difference
PCA: Posterior condylar axis

## References

[1] Berger RA, Crossett LS, Jacobs JJ, Rubash HE (1998). Malrotation causing patellofemoral complications after total knee arthroplasty. Clin Orthop.

[2] Jeffery RS, Morris RW, Denham RA (1991). Coronal alignment after total knee replacement. J Bone Joint Surg Br.

[3] Kandemir U, Yazici M, Alpaslan AM, Surat A (2002). Morphology of the knee in adult patients with neglected developmental dysplasia of the hip. J Bone Joint Surg Am.

[4] Kettelkamp DB (1981). Management of patellar malalignment. J Bone Joint Surg Am.

[5] Matsuda S, Miura H, Nagamine R, Mawatari T, Tokunaga M, Nabeyama R (2004). Anatomical analysis of the femoral condyle in normal and osteoarthritic knees. J Orthop Res.

[6] Minoda Y, Kobayashi A, Iwaki H, Sugama R, Iwakiri K, Kadoya Y (2008). Sagittal alignment of the lower extremity while standing in Japanese male. Arch Orthop Trauma Surg.

[7] Moreland JR, Bassett LW, Hanker GJ (1987). Radiographic analysis of the axial alignment of the lower extremity. J Bone Joint Surg Am.

[8] Watanabe S, Sato T, Omori G, Koga Y, Endo N (2014). Change in tibiofemoral rotational alignment during total knee arthroplasty. J Orthop Sci.

[9] Cooke TD, Li J, Scudamore RA (1994). Radiographic assessment of bony contributions to knee deformity. Orthop Clin North Am.

[10] Hsu RW, Himeno S, Coventry MB, Chao EY (1990). Normal axial alignment of the lower extremity and load-bearing distribution at the knee. Clin Orthop.

[11] Kawakami H, Sugano N, Yonenobu K, Yoshikawa H, Ochi T, Hattori A (2004). Effects of rotation on measurement of lower limb alignment for knee osteotomy. J Orthop Res.

[12] Akagi M, Mori S, Nishimura S, Asano T, Hamanishi C (2005). Variability of extraarticular tibial rotation references for total knee arthroplasty. Clin Orthop Relat Res.

[13] Ariumi A, Sato T, Kobayashi K, Koga Y, Omori G, Minato I (2010). Three-dimensional lower extremity alignment in the weight-bearing standing position in healthy elderly subjects. J Orthop Sci.

[14] Imai N, Ito T, Takahashi Y, Horigome Y, Suda K, Miyasaka D (2013). In vivo relationship between the clinical epicondylar axis and the anterior pelvic plane in normal subjects. J Biomed Sci Eng.

[15] Imai N, Miyasaka D, Ito T, Suzuki H, Minto I, Endo N (2017). The anteroposterior axis of the tibia is approximately perpendicular to the anterior pelvic plane in the standing position in healthy Japanese subjects. J Orthop Surg Res.

[16] Sugano N, Noble PC, Kamaric E, Salama JK, Ochi T, Tullos HS (1998). The morphology of the femur in developmental dysplasia of the hip. J Bone Joint Surg (Br).

[17] Noble PC, Sugano N, Kamaric E, Matsubara M, Harada Y, Ohzono K (2003). The three-dimensional shape of the dysplastic femur: implications for THR. Clin Orthop Relat Res.

[18] Reikeras O, Bjerkreim I, Kolbenstvedt A (1982). Ideopathic increased anteversion of the acetabulum and femoral neck. Acta Orthop Scand.

[19] Agarwal A, Gupta N (2012). Risk factor and diagnosis of developmental dysplasia of hip in children. J Clin Orthop Trauma.

[20] Naito M, Nakamura Y (2014). Curved periacetabular osteotomy for treatment of dysplastic hip. Clin Orthop Surg.

[21] Sato T, Koga Y, Omori G (2004). Three-dimensional lower extremity alignment assessment system: application to evaluation of component position after total knee arthroplasty. J Arthroplast.

[22] Kobayashi K, Sakamoto M, Tanabe Y, Ariumi A, Sato T, Omori G (2009). Automated image registration for assessing three-dimensional alignment of entire lower extremity and implant position using bi-plane radiography. J Biomech.

[23] Lewinnek GE, Lewis JL, Tarr R, Compere CL, Zimmerman JR (1978). Dislocations after total hip-replacement arthroplasties. J Bone Joint Surg.

[24] Nakahara I, Takao M, Sakai T, Nishii T, Yoshikawa H, Sugano N (2011). Gender differences in 3D morphology and bony impingement of human hips. J Orthop Res.

[25] Sugano N, Noble PC, Kamaric E (1998). A comparison of alternative methods of measuring femoral anteversion. J Comput Assist Tomogr.

[26] Parikh S, Frank R, Noyes FR (2011). Patellofemoral disorders: role of computed tomography and magnetic resonance imaging in defining abnormal rotational lower limb alignment. Sports Health.

[27] Churchill DL, Incavo SJ, Johnson CC, Beynnon BD (1998). The transepicondylar axis approximates the optimal flexion axis of the knee. Clin Orthop Relat Res.

[28] Johal P, Williams A, Wragg P, Hunt D, Gedroyc W (2005). Tibio-femoral movement in the living knee. A study of weight bearing and non-weight bearing knee kinematics using “interventional” MRI. J Biomech.

[29] Kozanek M, Hosseini A, Liu F, Van de Velde SK, Gill TJ, Rubash HE (2009). Tibiofemoral kinematics and condylar motion during the stance phase of gait. J Biomech.

[30] Chen HN, Yang K, Dong QR, Wang Y (2014). Assessment of tibial rotation and meniscal movement using kinematic magnetic resonance imaging. J Orthop Surg Res.

